# Prevalence of Low Back Pain Among University Attendants in Tabuk City During 2023: A Cross-Sectional Study in Saudi Arabia

**DOI:** 10.7759/cureus.50357

**Published:** 2023-12-11

**Authors:** Areej A Aljohani, Saleem M Alarawi, Yasir M Alhusayni, Reema A Alanazi, Amira A Alkonani, Bedour E Alatawi, Ishtiyaq A Abu Zayied, Maram K Alshammari, Amal S Alhawaiti, Sarah A Abu Sabir, Amirah A Alatawi

**Affiliations:** 1 Faculty of Medicine, University of Tabuk, Tabuk, SAU; 2 Family Medicine, Prince Sultan Armed Forces Hospital, Medina, SAU

**Keywords:** risk factor, prevalence, oswestry low back pain disability score, medical student, low back pain

## Abstract

Background: College students are at a higher risk of suffering low back pain (LBP). Assessing the magnitude of the problem and the associated risk factors can help reduce the suffering and disability in future doctors.

Aim: This study was conducted to investigate the prevalence and related factors of LBP among medical students in the University of Tabuk and emphasize the need for targeted interventions that could help alleviate the burden of LBP among the students and improve their quality of life.

Methods: This cross-sectional study used an online well-structured, self-report questionnaire to collect the respondents’ data. The questionnaire explored the participants’ sociodemographic factors, lifestyle, and the severity of LBP-related disability using the modified Oswestry Low Back Pain Disability Questionnaire (Oswestry Disability Index (ODI)) score.

Results: The prevalence rate of LBP was 26.8%. The independent factors that significantly increased the probability of LBP included overweight/obesity (odds ratio (OR): 1.696, 95% confidence interval (CI): 1.086 to 2.648, p = 0.020) and stretching exercises (OR: 1.784, 95% CI: 1.104 to 2.883, p = 0.018). The independent predictors that significantly increased the severity of ODI included married marital status (p = 0.007), back surgery (p = 0.031), and higher pain intensity (p < 0.001).

Conclusions: We found that the prevalence rate of LBP among our sample was around 26%. This rate is approximate to the rates reported in previous studies. Furthermore, the activities most affected by LBP were sitting, standing, and lifting. Future studies should explore other risk factors and attempt to determine the onset of pain. A longitudinal study design is recommended to identify the onset of developing LBP.

## Introduction

Low back pain (LBP) is considered one of the most common musculoskeletal disorders globally. It is the leading cause of disability worldwide and affects people of all ages and professions [1]. LBP is defined as "pain in the area on the posterior aspect of the body from the lower margin of the twelfth ribs to the lower gluteal folds with or without pain referred into one or both lower limbs that lasts for at least one day" [2,3]. Medical students in particular are at a high risk for developing LBP because of the demanding nature of their curriculum [4]. Many modifiable risk factors were associated with LBP among medical students, such as a sedentary lifestyle, poor body posture, and prolonged standing or sitting, which can aid in identifying effective interventions for managing LBP to improve the quality of life and academic performance of medical students [4].

A medical student spends prolonged hours studying, performing different clinical rotations for hours per day, and attending lectures, which can result in prolonged standing or sitting and awkward different body positions. The incidence of LBP among medical students has been reported to increase with academic progression during medical school by up to 75% [5,6]. LBP has a negative impact on medical students and their academic performance, mental health, or even career prospects. Medical students experiencing LBP may experience reduced concentration, impaired physical activity, and poor sleep quality, leading to decreased academic performance and increased stress levels [5,6]. Sitting for more than eight hours and lacking physical exercise were found to be independently associated with a higher prevalence of LBP [7,8]. It is a major contributor to occupational disability globally and the main reason people miss work. This condition is not likely to change as the population ages due to adopting a more sedentary lifestyle [9]. 

Owing to the lack of this type of study among medical students in Tabuk City, Saudi Arabia, our study aims to investigate the prevalence and related factors of LBP among medical students in Tabuk City and emphasize the need for targeted interventions that could help alleviate the burden of LBP among medical students and improve their quality of life.

This article was previously presented as a poster at The Annual Research Forum at the Faculty of Medicine, University of Tabuk, on August 31, 2023.

## Materials and methods

Study design

This is a cross-sectional observational study. The participants are exclusively undergraduate medical students from the University of Tabuk, Saudi Arabia, from the preparatory year till the internship, which is the last year.

Study setting

The study was conducted at the Faculty of Medicine at the University of Tabuk, Tabuk City, Saudi Arabia. The University of Tabuk is a governmental institution and is considered a major university in the Tabuk region. 

Study population and targeted group

The inclusion criteria include (i) undergraduate medical students of the Faculty of Medicine at the University of Tabuk and (ii) male and female genders. 

The exclusion criteria include (i) postgraduates; (ii) students from other health specialities, such as pharmacy, nursing, medical laboratories, and others; (iii) employees of the University of Tabuk; and (iv) the teaching staff of the University of Tabuk.

Sample size 

The initial sample size was 391, which was calculated with an estimated prevalence of 25.6%, with 95% confidence interval (CI), and maximally 10 predictors for binomial logistic regression. We added 20% to allow for missing data, so the final sample size was 470 participants.

Data collection tool

Structured, anonymous, self-report questionnaires were applied for data collection. The content was adopted from previous literature and revised by family medicine consultants for validity, with the addition of refusal demographic data and implementation of the Oswestry Disability Index (ODI). A pilot test was performed on a small group of university students to test the clarity, ease, and time needed to complete the questionnaire. The questionnaire required about 10 minutes to be completed. The final version of the questionnaire was developed electronically through Google Forms (Google, USA). 

Statistical analysis

Statistical analysis was carried out using the IBM SPSS Statistics for Windows, version 28 (released 2021; IBM Corp., Armonk, New York, United States). Categorical variables were presented as counts and percentages. Numerical variables were presented as the mean, standard deviation (SD), or median and interquartile range (25th-75th percentiles). The associations between LBP and relevant nominal variables were assessed using the Pearson’s chi-squared test for independence of observations, Fisher’s exact test, and Fisher-Freeman-Halton exact test. The associations between LBP and ordinal variables were done using the Chocran-Armitage test for trend. Binomial logistic regression and linear regression analyses were carried out to assess the independent factors affecting LBP and the severity of the ODI score. Univariate analysis was first performed; then, relevant variables with a p < 0.001 were entered into the multivariate analysis. Statistical significance was considered at p < 0.05. 

## Results

Sociodemographic characteristics of all respondents

A total of 470 online questionnaires were distributed, out of which 451 were completed with a response rate of 96%. Most respondents (66.5%) belonged to the age group of 18-22 years, followed by the age group of 23-25 years (28.4%). Most respondents were females (68.1%), and around one-third of the respondents were overweight or obese. Only 10.6% of the respondents suffered from comorbidities. Most respondents were single (97.3%). The largest rate of responses came from students in the third (20.2%), fourth (28.8%), and fifth (21.7%) academic years, as represented in Table 1.

**Table 1:** Sociodemographic characteristics of the studied respondents (total N = 451) FE: Fisher’s exact test; N: number; X^2_ChS_^: Pearson’s chi-square test for independence of observations; X^2^_FFH_: Fisher-Freeman-Halton exact test; X^2^_L_: Chi-square test for linear-by-linear association test (Cochran-Armitage test for trend); * significant at p<0.05

Respondents’ characteristics		Total (N = 451)	No LBP (N = 330)	LBP (N = 121)	Test statistic	p-value
Age (years)	18-22	300 (66.5%)	229 (69.4%)	71 (58.7%)	X^2^_L_ = 5.146	0.026*
	23-25	128 (28.4%)	87 (26.4%)	41 (33.9%)		
	26-20	21 (4.7%)	13 (3.9%)	8 (6.6%)		
	=>30	2 (0.4%)	1 (0.3%)	1 (0.8%)		
Gender	Female	307 (68.1%)	225 (68.2%)	82 (67.8%)	X^2^_ChS_ = 0.007	0.934
	Male	144 (31.9%)	105 (31.8%)	39 (32.2%)		
BMI	Underweight (<18.5)	47 (10.4%)	39 (11.8%)	8 (6.6%)	X^2^_L_ = 6.633	0.010*
	Healthy weight (18.5-24.9)	248 (55.0%)	188 (57.0%)	60 (49.6%)		
	Overweight (25.0-29.9)	107 (23.7%)	71 (21.5%)	36 (29.8%)		
	Obesity (=>30)	49 (10.9%)	32 (9.7%)	17 (14.0%)		
Comorbidities	No	403 (89.4%)	296 (89.7%)	107 (88.4%)	X^2^_ChS_ = 0.149	0.699
	Yes	48 (10.6%)	34 (10.3%)	14 (11.6%)		
Present comorbidities	asthma	28 (6.2%)	20 (6.1%)	8 (6.6%)	X^2^_ChS_ = 0.046	0.830
	DM	7 (1.6%)	5 (1.5%)	2 (1.7%)	FE	1.000
	Hypertension	5 (1.1%)	4 (1.2%)	1 (0.8%)	FE	1.000
	IBS	2 (0.4%)	2 (0.6%)	0 (0.0%)	FE	1.000
	hypothyroidism	2 (0.4%)	1 (0.3%)	1 (0.8%)	FE	0.465
	hyperlipidemia	1 (0.2%)	1 (0.3%)	0 (0.0%)	FE	1.000
	Wilson	1 (0.2%)	0 (0.0%)	1 (0.8%)	FE	0.268
	Allergic rhinitis	1 (0.2%)	1 (0.3%)	0 (0.0%)	FE	1.000
	PCOS	1 (0.2%)	1 (0.3%)	0 (0.0%)	FE	1.000
	eczema	1 (0.2%)	1 (0.3%)	0 (0.0%)	FE	1.000
	psoriasis	1 (0.2%)	0 (0.0%)	1 (0.8%)	FE	0.268
Marital status	Divorced	3 (0.7%)	0 (0.0%)	3 (2.5%)	X^2^_FFH_ = 8.301	0.008*
	Married	9 (2.0%)	5 (1.5%)	4 (3.3%)		
	Single	439 (97.3%)	325 (98.5%)	114 (94.2%)		
Academic year	1st	20 (4.4%)	16 (4.8%)	4 (3.3%)	X^2^_L_ = 4.165	0.041*
	2nd	36 (8.0%)	23 (7.0%)	13 (10.7%)		
	3rd	91 (20.2%)	75 (22.7%)	16 (13.2%)		
	4th	130 (28.8%)	102 (30.9%)	28 (23.1%)		
	5th	98 (21.7%)	64 (19.4%)	34 (28.1%)		
	6th	34 (7.5%)	24 (7.3%)	10 (8.3%)		
	Internship	42 (9.3%)	26 (7.9%)	16 (13.2%)		

Characteristics of the lifestyle factors of all respondents

As regard the practice of sports, 43.5% of the respondents did not do any practicing, while 44.1% exercised for one to three days/week and 12.4% for four to seven days/week. Nearly one-quarter of the respondents do exercises for 30-60 minutes per session. The most common type of exercise was cardio exercises (37.7%), followed by stretching exercises (11.1%), resistance exercises (25.1%), and, lastly, sports competitions (11.1%). Considering the respondents' usual food patterns, healthy eating was "always relied on" (9.8%), "sometimes consumed" (47.5%), "rarely eaten" (24.8%), and "not relied on" (18.0%). Only 11.8% of the respondents were smokers, as shown in Table 2.

**Table 2:** Lifestyle characteristics of the studied respondents (total N = 451) N: number; X^2^_ChS_: Pearson’s chi-square test for independence of observations; X^2^_L_: chi-square test for linear-by-linear association test (Cochran-Armitage test for trend); * significant at p<0.05

Lifestyle factors		Total (N = 451)	No LBP (N = 330)	LBP (N = 121)	Test statistic	p-value
How many days on average do you exercise in a week?	I do not do sports.	196 (43.5%)	146 (44.2%)	50 (41.3%)	X^2^_L_ = 0.309	0.578
	1-3 days a week	199 (44.1%)	144 (43.6%)	55 (45.5%)		
	4-7 days a week	56 (12.4%)	40 (12.1%)	16 (13.2%)		
How long does your exercise session usually take?	I do not do exercises.	196 (43.5%)	146 (44.2%)	50 (41.3%)	X^2^_L_ = 1.142	0.289
	Less than 30 minutes	67 (14.9%)	50 (15.2%)	17 (14.0%)		
	30-60 minutes	112 (24.8%)	81 (24.5%)	31 (25.6%)		
	1-2 hours	66 (14.6%)	49 (14.8%)	17 (14.0%)		
	More than two hours	10 (2.2%)	4 (1.2%)	6 (5.0%)		
What kind of exercises do you do in one exercise session?	Cardio exercises	170 (37.7%)	123 (37.3%)	47 (38.8%)	X^2^_ChS_=0.093	0.760
	Resistance (strength) exercises	113 (25.1%)	77 (23.3%)	36 (29.8%)	X^2^_ChS_=1.943	0.163
	Sports competitions	50 (11.1%)	37 (11.2%)	13 (10.7%)	X^2^_ChS_=0.020	0.888
	Stretching exercises	114 (25.3%)	76 (23.0%)	38 (31.4%)	X^2^_ChS_=3.288	0.070
How many days per week do you stretch (stretch)?	I do not do stretching exercises.	332 (73.6%)	250 (75.8%)	82 (67.8%)	X^2^_L_ = 1.679	0.195
	1-3 days a week	99 (22.0%)	65 (19.7%)	34 (28.1%)		
	4-7 days a week	20 (4.4%)	15 (4.5%)	5 (4.1%)		
What is your usual food pattern?	I rely on healthy eating permanently	44 (9.8%)	36 (10.9%)	8 (6.6%)	X^2^_L_ = 0.456	0.499
	I rely on healthy eating sometimes	214 (47.5%)	145 (43.9%)	69 (57.0%)		
	I rarely eat healthy food	112 (24.8%)	88 (26.7%)	24 (19.8%)		
	I do not rely on healthy eating	81 (18.0%)	61 (18.5%)	20 (16.5%)		
Are you a smoker?	No	398 (88.2%)	297 (90.0%)	101 (83.5%)	X^2^_ChS_=3.639	0.056
	Yes	53 (11.8%)	33 (10.0%)	20 (16.5%)		

Prevalence rate and characteristics of LBP

**Figure 1:**
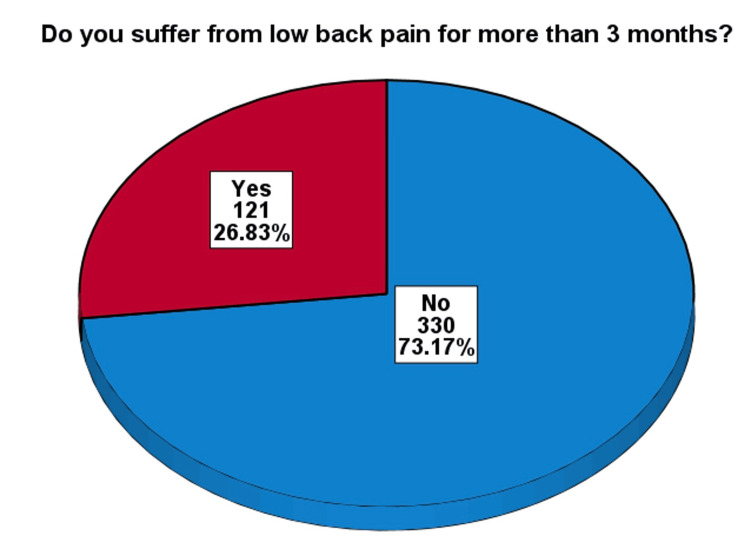
Prevalence of participants with low back pain for more than three months (total N = 451)

In Figure 1, out of the 451 respondents, 121 (26.8%) had LBP for more than three months.

The duration of pain was three to six months in 47.1%, six to 12 months in 24%, one to two years in 18.2%, two to four years in 5%, and above four years in 5.8%. Most respondents with LBP described the pain as irregular (86.8%) and had no apparent cause to explain the pain (89.3%). Only 5.8% of those with LBP had or were planning to have back surgery. Only two out of the 82 females with LBP were pregnant (2.4%), as shown in Table 3.

**Table 3:** Pain characteristics in respondents with low back pain for more than three months (N = 121)

Pain characteristics		N (%)
What is the approximate duration ?	3-6 months	57 (47.1%)
	6-12 months	29 (24.0%)
	One to two years	22 (18.2%)
	2 to 4 years	6 (5.0%)
	More than 4 years	7 (5.8%)
How would you describe the low back pain you are experiencing?	Irregular pain	105 (86.8%)
	Regular pain	16 (13.2%)
Is the cause of your low back pain due to an injury/accident, infection, tumor, rheumatoid arthritis, or a congenital anomaly?	No	108 (89.3%)
	Yes	13 (10.7%)
Have you had back surgery before, or will you have it soon?	No	114 (94.2%)
	Yes	7 (5.8%)
If you are female, are you pregnant? (N=82)	No	80 (97.6%)
	Yes	2 (2.4%)

**Figure 2:**
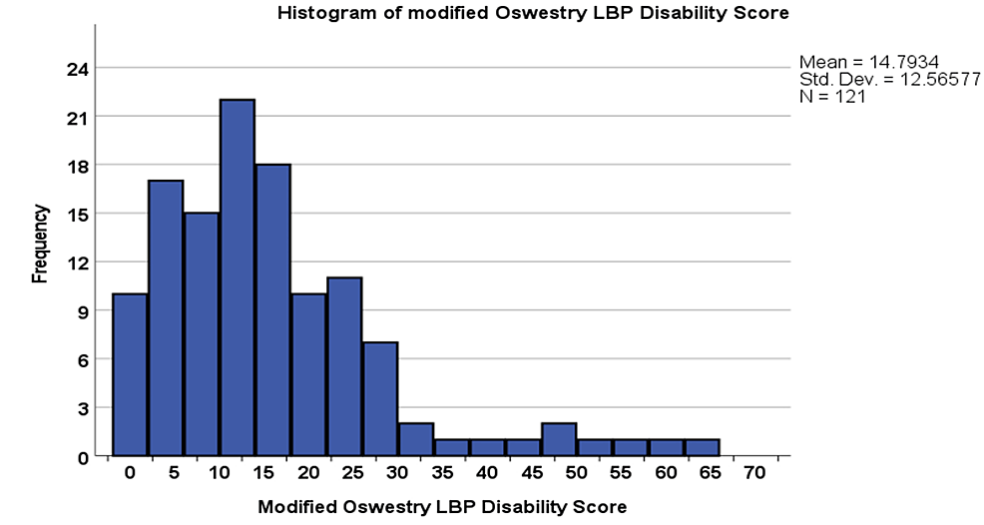
Histogram of modified Oswestry Low Back Pain Disability Questionnaire score in respondents with low back pain (N = 121)

In response to the modified Oswestry Low Back Pain Disability Questionnaire tool, the most affected domains were sitting (1.19 ± 1.11), standing (1.19 ± 1.06), and lifting (0.94 ± 0.93) (Table 4). The overall modified Oswestry Low Back Pain Disability Questionnaire score was 14.79 ± 12.66 (range: 0-62) (Figure 2).

**Table 4:** Responses of the respondents with low back pain to modified Oswestry Low Back Pain Disability Questionnaire (N = 121) IQR: interquartile range (expressed as 25th–75th percentiles); SD: standard deviation; 0 indicates the absence of effect of pain, while 5 indicates the severest degree of disability in each section.

	0	1	2	3	4	5	Mean ± SD	Median (IQR)
1 – Pain intensity	69 (57.0%)	27 (22.3%)	17 (14.0%)	4 (3.3%)	2 (1.7%)	2 (1.7%)	0.75 ± 1.10	0 (0-1)
2 – Personal care	92 (76.0%)	22 (18.2%)	3 (2.5%)	2 (1.7%)	2 (1.7%)	0 (0.0%)	0.35 ± 0.76	0 (0-0)
3 – Lifting	39 (32.2%)	63 (52.1%)	9 (7.4%)	8 (6.6%)	1 (0.8%)	1 (0.8%)	0.94 ± 0.93	1 (0-1)
4 – Walking	84 (69.4%)	24 (19.8%)	6 (5.0%)	5 (4.1%)	2 (1.7%)	0 (0.0%)	0.49 ± 0.90	0 (0-1)
5 – Sitting	38 (31.4%)	39 (32.2%)	34 (28.1%)	5 (4.1%)	3 (2.5%)	2 (1.7%)	1.19 ± 1.11	1 (0-2)
6 – Standing	31 (25.6%)	55 (45.5%)	23 (19.0%)	6 (5.0%)	5 (4.1%)	1 (0.8%)	1.19 ± 1.06	1 (0-2)
7 – Sleeping	87 (71.9%)	21 (17.4%)	7 (5.8%)	4 (3.3%)	1 (0.8%)	1 (0.8%)	0.46 ± 0.91	0 (0-1)
8 – Social life	79 (65.3%)	26 (21.5%)	6 (5.0%)	6 (5.0%)	3 (2.5%)	1 (0.8%)	0.60 ± 1.05	0 (0-1)
9 – Travel/commute	66 (54.5%)	44 (36.4%)	4 (3.3%)	3 (2.5%)	2 (1.7%)	2 (1.7%)	0.65 ± 0.99	0 (0-1)
10 – Job/housework	57 (47.1%)	46 (38.0%)	12 (9.9%)	2 (1.7%)	3 (2.5%)	1 (0.8%)	0.77 ± 0.97	1 (0-1)

Association of LBP with sociodemographic and lifestyle characteristics

The comparison between respondents with and without LBP showed a significant tendency for LBP in older age groups (p = 0.026), overweight and obese respondents (p = 0.010), and higher academic years (p = 0.041). In addition, more married and divorced respondents had LBP (p = 0.008). There were no significant differences between the two groups regarding gender or comorbidities (all p < 0.05; Table 1). Moreover, no significant differences were found regarding the type and frequency of exercises, healthy eating, and smoking (all p < 0.05; Table 2). 

Multivariate logistic regression analysis was conducted to assess the independent factors that increased or decreased the likelihood of LBP. Variables that were eligible for the model included age, overweight/obesity, academic year, stretching exercises, and smoking. The independent factors that increased the probability of having LBP included overweight/obesity (OR: 1.696; 95% CI: 1.086 to 2.648; p = 0.020) and stretching exercises (OR: 1.784; 95% CI: 1.104 to 2.883; p = 0.018; Table 5).

**Table 5:** Logistic regression analysis for factors increasing the likelihood of low back pain in the studied sample ( N = 451) B: unstandardized regression coefficient; CI: confidence interval; OR: odds ratio; SE: standard error for B: significant at p<0.05

Independent factors	B	S.E.	p-value	OR	95% C.I. for OR
Univariate analysis					
Age (years)	0.382	0.170	0.025*	1.465	1.050 to 2.042
Male gender (ref = female)	0.019	0.228	0.934	1.019	0.652 to 1.592
Overweight & Obesity (ref = non-overweight/non-obese)	0.541	0.218	0.013*	1.718	1.120 to 2.635
Presence of comorbidities (reference = absent)	0.130	0.337	0.699	1.139	0.588 to 2.205
Married marital status (ref = single/divorced)	0.799	0.679	0.240	2.222	0.587 to 8.416
Academic year	0.139	0.068	0.042*	1.149	1.005 to 1.313
How many days on average do you exercise in a week?	0.086	0.155	0.578	1.090	0.804 to 1.479
How long does your exercise session usually take?	0.094	0.088	0.285	1.098	0.925 to 1.305
Cardio exercises	0.067	0.219	0.760	1.069	0.697 to 1.640
Resistance (strength) exercises	0.330	0.238	0.164	1.392	0.873 to 2.217
Sports competitions	-0.048	0.342	0.888	0.953	0.488 to 1.862
Stretching exercises	0.425	0.235	0.071	1.530	0.964 to 2.428
How many days per week do you stretch?	0.240	0.186	0.196	1.272	0.883 to 1.831
What is your usual food pattern?	-0.081	0.119	0.499	0.923	0.730 to 1.166
Smoker (ref = non-smoker)	0.578	0.306	0.059	1.782	0.979 to 3.246
Multivariate analysis					
Age (years)	0.237	0.214	0.268	1.268	0.833 to 1.928
Overweight & Obesity (ref = non-overweight/non-obese)	0.528	0.227	0.020*	1.696	1.086 to 2.648
Academic year	0.060	0.083	0.472	1.062	0.902 to 1.250
Stretching exercises	0.579	0.245	0.018*	1.784	1.104 to 2.883
Smoker (ref = non-smoker)	0.520	0.315	0.099	1.682	0.906 to 3.121

Association of the modified Oswestry Low Back Pain Disability Questionnaire score with sociodemographic, lifestyle, and pain characteristics

Univariate linear regression analysis was carried out to assess the factors that significantly affected the overall score. Higher scores were significantly associated with married marital status (B = 14.692; 95% CI: 2.271 to 27.114; p = 0.021), increased frequency of doing stretching exercises per week (B = 4.081; 95% CI: 0.096 to 8.066; p = 0.045), and increased pain severity (B = 2.596; 95% CI: 1.505 to 3.687; p < 0.001). Sports competitions were associated with higher scores, but the p-value was slightly above the significance level (B = 6.350; 95% CI: -0.893 to 13.594; p = 0.085). These factors were eligible for inclusion in the multivariate model, and the independent predictors increasing the severity of disability included married marital status (B = 15.388; 95% CI: 4.271 to 26.504; p = 0.007), back surgery (B = 9.505; 95% CI: 0.909 to 18.102; p = 0.031), and increased pain intensity (B = 2.431; 95% CI: 1.378 to 3.484; p < 0.001; Table 6).

**Table 6:** Linear analysis for factors affecting the modified Oswestry Low Back Pain Disability Questionnaire score (N = 121) B: unstandardized regression coefficient; CI: confidence interval; SE: standard error for B: significant at p < 0.05

Independent factors	B	S.E.	p-value	95% C.I. for B
Univariate analysis				
Age (years)	0.543	1.745	0.756	-2.912 to 3.999
Gender	1.251	2.452	0.611	-3.604 to 6.106
Overweight and obesity	-1.680	2.307	0.468	-6.248 to 2.888
Comorbidities	5.080	3.556	0.156	-1.961 to 12.121
Married marital status	14.692	6.273	0.021*	2.271 to 27.114
Academic year	0.039	0.690	0.955	-1.328 to 1.406
How many days on average do you exercise in a week?	0.230	1.679	0.891	-3.096 to 3.555
How long does your exercise session usually take?	1.473	0.896	0.103	-0.301 to 3.247
Cardio exercises	-2.828	2.339	0.229	-7.460 to 1.804
Resistance (strength) exercises	1.085	2.507	0.666	-3.880 to 6.050
Sports competitions	6.350	3.658	0.085	-0.893 to 13.594
Stretching exercises	1.912	2.465	0.439	-2.969 to 6.794
How many days per week do you stretch?	4.081	2.013	0.045*	0.096 to 8.066
What is your usual food pattern?	0.460	1.359	0.736	-2.232 to 3.151
Are you a smoker?	4.201	3.064	0.173	-1.866 to 10.268
What is the approximate duration?	0.708	0.977	0.470	-1.227 to 2.644
Have you had back surgery before, or will you have it soon?	10.985	4.809	0.024*	1.462 to 20.508
The pain intensity scale	2.596	0.551	<0.001*	1.505 to 3.687
Multivariate analysis				
Married marital status	15.388	5.612	0.007*	4.271 to 26.504
Sports competitions	5.243	3.332	0.118	-1.357 to 11.843
How many days per week do you stretch?	1.910	1.854	0.305	-1.763 to 5.584
Have you had back surgery before, or will you have it soon?	9.505	4.340	0.031*	0.909 to 18.102
The pain intensity scale	2.431	0.531	<0.001*	1.378 to 3.484

## Discussion

Medical students are at risk of suffering LBP due to the nature of their studies requiring long hours of sitting for studying, which favors a sedentary lifestyle and reduces the opportunities for physical exercise. In addition, the experienced stress of frequent examinations and encounters with patients contribute to increasing the risk of LBP among medical students [10-15]. The present study aimed to investigate the prevalence and related factors of LBP among medical students in Tabuk City and emphasize the need for targeted interventions that could help alleviate the burden of LBP among medical students and improve their quality of life.

We found that the prevalence rate of LBP among our sample was 26.8%. This rate is approximate to the rates reported in previous studies. In Saudi Arabia, the rate of LBP was 23.2% among five health sciences colleges [16] and 33.3% among Taif medical students [17]. In addition, the prevalence rate of LBP was 20.8% in Serbia [18], 27.2% in Malaysia [19], and 25.6% in Pakistan [20]. Meanwhile, much lower rates were reported by previous studies in Brazil (9.2%) [21] and Pakistan (13.0%) [22]. Higher rates of LBP among medical students were reported from Saudi Arabia (33.4% to 40.5%) [10,15], India (32.5%) [23], Turkey (34.6%) [24], Pakistan (38.6%) [25], and Serbia (59.5%) [26]. The discrepancy in the reported rates of LBP in the previous studies could be explained by variations in the study designs, the used questionnaires, the curricula of medical colleges, and the basic characteristics of the populations from which the samples were drawn.

In the current study, the activities most affected by LBP were sitting, standing, and lifting. The overall modified ODI score ranged between 0 and 62 with an average of 14.79 ± 12.66.

The current study attempted to identify the potential risk factors that affect the probability of developing LBP or increasing LBP-related disability. The identification of these factors is important to design effective strategies that help medical students to reduce the risk or severity of LBP.

We found that students with LBP significantly tended to be older, but this effect was non-significant in the multivariate model. As regard the ODI score within those with LBP, age had no significant impact. Several studies have stated that the prevalence of LBP increases with age in the univariate analysis [17,26], but one study [17] was similar to our finding, such that the association was not significant in the multivariate regression. Meanwhile, other studies found a lack of significant association between age and LBP [6,20,18,27,28]. The assumed effect of age is attributed to the increased duration of exposure to stress at the college, particularly when clinical years are approached.

As regard the BMI, LBP was significantly associated with overweight/obesity in the current study in both the univariate and multivariate analyses. Meanwhile, overweight/obesity did not significantly affect the severity of the ODI score in those with LBP. This finding is supported by previous studies, which reported a significantly higher prevalence in those with LBP in univariate analysis [4] and multivariate regression [20]. This effect of BMI could be explained by the increased pressure on the spine structures with the increase in body weight, which will induce back pain [29]. On the contrary, some studies [5,17,18,24,26,28] did not find an association of LBP with BMI in the univariate analysis.

In this study, students with LBP significantly tended to be in higher academic years in univariate analysis, but this significant association was not observed in adjusting for other factors. Moreover, the ODI score did not correlate with the academic years. In partial agreement with these findings, Falavigna et al. [21], Amelot et al. [5], and Alturkistani et al. [17] found that participants with LBP were significantly more prevalent in higher academic years in the univariate and multivariate analyses. However, other studies [4,6,18,27] found no significant association of LBP with academic years. Higher academic years are usually more stressful to medical students as the quantity of the curriculum is increased besides the demands for intense clinical training.

In the current study, gender had no significant impact on the probability of LBP or the severity of disability, which was agreed by multiple studies [4,5,18,23]. On the other hand, LBP was significantly associated with female gender in previous studies in univariate [5,27,28] and multivariate analyses [17,20,27]. 

Marital status can hypothetically affect the probability of LBP by increasing the individual’s responsibilities, thus increasing stress. Moreover, married women may be at a higher risk of LBP due to pregnancy and the physical activities associated with caring for their offspring and their homes. We found that students with LBP significantly tended to be married or divorced, but the association was not significant when tested using univariate logistic regression. The marital status significantly increased the severity of disability compared to the single and divorced participants in univariate and multivariate regression. Similarly, Alturkistani et al. did not find a significant difference in marital status between students with and without LBP.

The results of our study showed that comorbidities were not associated with LBP or affected the severity of disability. This lack of association with LBP occurrence accords with the results of previous studies on medical students [18]. This may be explained by the low number of comorbidities in this age group, which comprised mainly asthma and other illnesses not likely to increase the risk of LBP.

Although several studies recommended physical exercise as a protective factor to reduce the risk of LBP [30], we found that the type of exercise had no significant impact on the presence of LBP or the severity of the disability. The frequency of stretching exercises per week increased the ODI score in univariate linear regression but not in multivariate regression. Similarly, several studies did not reveal any significant association between physical activity and LBP prevalence among medical students [21-24]. However, some other studies reported a significant relationship between LBP and the frequency of physical activity [5,20,26,27]. The lack of protective effect of exercise in the present study may partially be attributed to the low participation in sports and physical activity, which was observed in nearly half of the respondents. 

We found that smoking neither affected the probability of LBP nor the severity of the disability. This accords with numerous previous studies, which found a lack of association between smoking and LBP [15,16,23, 25,27]. However, cigarette smoking was significantly associated with a higher rate of LBP according to the results of other studies [18,20,22]. This controversy may be attributed to the low number of smokers in our sample, which was expected as female students constituted nearly two-thirds of the sample. The prevalence of smoking among females in Arab countries is comparatively lower than that in European and American countries. Younger age (below 22 years) may play a role as smoking is less prevalent in this age group [18].

## Conclusions

The present study identified the prevalence rate of LBP during the three months preceding the study, which was 26.8%. This rate was comparable to other studies, although lower and higher rates were reported previously. The independent factors that significantly increased the probability of LBP included overweight/obesity and stretching exercises. The independent predictors that significantly increased the severity of disability included married marital status, back surgery, and higher pain intensity. Some of these factors are modifiable and can be controlled to reduce the prevalence of LBP, such as obesity. We recommend that future studies should explore other risk factors and attempt to determine the onset of pain. Moreover, studies can be longitudinal in design and enroll students from the first year at the college till their graduation.

## References

[1] Hoy D, Brooks P, Blyth F, Buchbinder R (2010). The Epidemiology of low back pain. Best Pract Res Clin Rheumatol.

[2] Hoy D, March L, Brooks P (2014). The global burden of low back pain: estimates from the Global Burden of Disease 2010 study. Ann Rheum Dis.

[3] Koes BW, van Tulder MW, Thomas S (2006). Diagnosis and treatment of low back pain. BMJ.

[4] Taha YA, Al Swaidan HA, Alyami HS (2023). The prevalence of low back pain among medical students: a cross-sectional study from Saudi Arabia. Cureus.

[5] Amelot A, Mathon B, Haddad R, Renault MC, Duguet A, Steichen O (2019). Low back pain among medical students: a burden and an impact to consider!. Spine (Phila Pa 1976).

[6] Tavares C, Salvi CS, Nisihara R, Skare T (2019). Low back pain in Brazilian medical students: a cross-sectional study in 629 individuals. Clin Rheumatol.

[7] Bontrup C, Taylor WR, Fliesser M, Visscher R, Green T, Wippert PM, Zemp R (2019). Low back pain and its relationship with sitting behaviour among sedentary office workers. Appl Ergon.

[8] Park JH, Srinivasan D (2021). The effects of prolonged sitting, standing, and an alternating sit-stand pattern on trunk mechanical stiffness, trunk muscle activation and low back discomfort. Ergonomics.

[9] Cohen SP, Argoff CE, Carragee EJ (2008). Management of low back pain. BMJ.

[10] Dighriri YH, Akkur MA, Alharbi SA, Madkhali NA, Matabi KI, Mahfouz MS (2019). Prevalence and associated factors of neck, shoulder, and low-back pains among medical students at Jazan University, Saudi Arabia: a cross-sectional study. J Family Med Prim Care.

[11] Edemekong PF, Bomgaars DL, Sukumaran S (2023). 2022 activities of daily living. StatPearls [Internet].

[12] Grabovac I, Dorner TE (2019). Association between low back pain and various everyday performances : activities of daily living, ability to work and sexual function. Wien Klin Wochenschr.

[13] Mlinac ME, Feng MC (2016). Assessment of activities of daily living, self-care, and independence. Arch Clin Neuropsychol.

[14] Rasmussen CD, Holtermann A, Jørgensen MB, Ørberg A, Mortensen OS, Søgaard K (2016). A multi-faceted workplace intervention targeting low back pain was effective for physical work demands and maladaptive pain behaviours, but not for work ability and sickness absence: Stepped wedge cluster randomised trial. Scand J Public Health.

[15] Algarni AD, Al-Saran Y, Al-Moawi A, Bin Dous A, Al-Ahaideb A, Kachanathu SJ (2017). The prevalence of and factors associated with neck, shoulder, and low-back pains among medical students at university hospitals in Central Saudi Arabia. Pain Res Treat.

[16] AlShayhan FA, Saadeddin M (2018). Prevalence of low back pain among health sciences students. Eur J Orthop Surg Traumatol.

[17] Alturkistani LH, Hendi OM, Bajaber AS, Alhamoud MA, Althobaiti SS, Alharthi TA, Atallah AA (2020). Prevalence of lower back pain and its relation to stress among medical students in Taif University, Saudi Arabia. Int J Prev Med.

[18] Ilic I, Milicic V, Grujicic S, Zivanovic Macuzic I, Kocic S, Ilic MD (2021). Prevalence and correlates of low back pain among undergraduate medical students in Serbia, a cross-sectional study. PeerJ.

[19] Alshagga MA, Nimer AR, Yan LP, Ibrahim IA, Al-Ghamdi SS, Radman Al-Dubai SA (2013). Prevalence and factors associated with neck, shoulder and low back pains among medical students in a Malaysian Medical College. BMC Res Notes.

[20] Sany SA, Tanjim T, Hossain MI (2021). Low back pain and associated risk factors among medical students in Bangladesh: a cross-sectional study. F1000Res.

[21] Falavigna A, Teles AR, Mazzocchin T (2011). Increased prevalence of low back pain among physiotherapy students compared to medical students. Eur Spine J.

[22] Hafeez K, Ahmed Memon A, Jawaid M, Usman S, Usman S, Haroon S (2013). Back pain - are health care undergraduates at risk?. Iran J Public Health.

[23] Aggarwal N, Anand T, Kishore J, Ingle GK (2013). Low back pain and associated risk factors among undergraduate students of a medical college in Delhi. Educ Health (Abingdon).

[24] Yucel H, Torun P (2016). Incidence and risk factors of low back pain in students studying at a health university. Bezmialem Sci.

[25] Haroon H, Mehmood S, Imtiaz F, Ali SA, Sarfraz M (2018). Musculoskeletal pain and its associated risk factors among medical students of a public sector University in Karachi, Pakistan. J Pak Med Assoc.

[26] Vujcic I, Stojilovic N, Dubljanin E, Ladjevic N, Ladjevic I, Sipetic-Grujicic S (2018). Low back pain among medical students in Belgrade (Serbia): a cross-sectional study. Pain Res Manag.

[27] Alrabai H, Aladhayani M, Alshahrani S, Alwethenani Z, Alsahil M, Algarni A (2021). Low back pain prevalence and associated risk factors among medical students at four major medical colleges in Saudi Arabia. J Nat Sci Med.

[28] Goweda RA, Idris KJ, Bakhsh AJ (2020). Prevalence and associated risk factor of low back pain among medical student of Umm Al- Qura University, Makkah, Saudi Arabia: cross-sectional study. Med Sci.

[29] Andrusaitis SF, Oliveira RP, Barros Filho TE (2006). Study of the prevalence and risk factors for low back pain in truck drivers in the state of São Paulo, Brazil. Clinics (Sao Paulo).

[30] Henchoz Y, Kai-Lik So A (2008). Exercise and nonspecific low back pain: a literature review. Joint Bone Spine.

